# Invasive Mucinous Adenocarcinoma Presenting as Persistent Bilateral Pulmonary Consolidations and Cavitation: A Diagnostic Challenge

**DOI:** 10.7759/cureus.117768

**Published:** 2026-10-11

**Authors:** Nasiba Hassan

**Affiliations:** 1 Internal Medicine, Great Western Hospital, Swindon, GBR

**Keywords:** bronchoscopy, ct-guided biopsy, granulomatous diseases, invasive mucinous adenocarcinoma of the lung, lung adenocarcinoma (ladc), never-smoker, pneumonic adenocarcinoma, pulmonary cavitation, pulmonary consolidation

## Abstract

Invasive mucinous adenocarcinoma (IMA) is an uncommon histological subtype of lung adenocarcinoma that can mimic pneumonia or other inflammatory lung diseases due to its distinct radiological features. Although multifocal consolidations, nodules, and masses are frequently observed, cavitation is relatively uncommon. These atypical radiological findings can complicate diagnosis and delay treatment. We present a case of a lifelong never-smoker in her late 60s who presented with chest pain and productive cough, a history of two recently treated chest infections, and significant weight loss. Laboratory evaluation showed elevated C-reactive protein (CRP) with normal procalcitonin and white blood cell count. Chest imaging revealed widespread bilateral consolidations, nodules, cavitation, and traction fibrosis, a pattern considered atypical for conventional lung malignancy and initially more suggestive of granulomatous disease or atypical infection. Extensive infectious, fungal, and autoimmune screening, along with serum angiotensin-converting enzyme level, did not identify an alternative cause. Bronchial wash and bronchoalveolar lavage (BAL) were non-diagnostic. Persistent symptoms and radiological abnormalities despite initial work-up ultimately led to CT-guided tissue biopsy of the lung, which confirmed the diagnosis of IMA. Further immunohistochemistry supported a primary pulmonary origin. Comprehensive molecular profiling did not reveal any clinically actionable driver alteration; therefore, she was commenced on first-line chemo-immunotherapy with carboplatin, pemetrexed, and pembrolizumab. She remains clinically stable and is tolerating treatment without dose-limiting toxicity; objective radiological response has not yet been assessed. This case highlights that persistent bilateral consolidations, nodules, cavitation, and fibrotic changes may occasionally represent an unusual presentation of lung malignancy, including IMA, even when infection or granulomatous disease is initially considered. Persistent radiological abnormalities despite negative microbiological and bronchoscopic investigations should prompt reconsideration of the differential diagnosis, even in lifelong never-smokers, with early tissue biopsy playing a pivotal role in establishing the diagnosis.

## Introduction

Invasive mucinous adenocarcinoma (IMA) is an uncommon histological subtype of lung adenocarcinoma, accounting for 3-10% of pulmonary adenocarcinomas [1]. IMA has distinct histological, radiological, and molecular features compared with conventional pulmonary adenocarcinoma. Pathological features of IMA include goblet or columnar tumour cells with abundant cytoplasmic mucin, and a lepidic growth pattern with microscopic skip lesions [1,2]. IMA also demonstrates a distinct molecular profile, with *KRAS* mutations being the most frequently identified driver alteration, while *NRG1* fusions represent an important recurrent genomic alteration [3]. Radiologically, IMA can present with nodular, consolidative, or pneumonic-like patterns, with ground-glass opacities, air bronchograms, and satellite nodules among the recognised findings [1,4]. The pneumonic-type presentation, characterised by multifocal or multilobar consolidation, can closely mimic inflammatory or infectious lung disease [1,4]. IMA may therefore initially be mistaken for pneumonia, tuberculosis, or other infectious or inflammatory lung diseases [5,6]. This diagnostic overlap can create uncertainty and potentially delay recognition of malignancy.

In our case, imaging showed widespread bilateral consolidations with multiple nodules, cavitation, and fibrotic changes with traction. Although IMA is recognised to present with multilobar consolidation, often accompanied by nodules or masses [1], cavitation is an uncommon radiological feature, with several mechanisms proposed, including ischaemic tumour necrosis, alveolar wall destruction by excessive mucus, and check-valve airway obstruction [2]. The presence of cavitation together with nodularity and traction/fibrotic change can therefore produce a radiological appearance that extends beyond the more typical pneumonia-like presentation of IMA and may broaden the differential diagnosis toward infection, granulomatous disease, or other inflammatory and fibrotic lung disorders.

We report a case of IMA in a lifelong never-smoking woman presenting with persistent bilateral pulmonary abnormalities initially considered more suggestive of atypical infection or granulomatous disease. Extensive microbiological and autoimmune investigations were unrevealing, while bronchoscopic sampling was non-diagnostic and repeat imaging showed persistent abnormalities without improvement. This subsequently prompted a CT-guided lung biopsy, which confirmed the diagnosis of IMA of the lung. This case highlights an atypical presentation of IMA in which cavitation and traction/fibrotic change accompanied the more recognised bilateral consolidative and nodular pattern, creating diagnostic overlap with infectious and granulomatous lung disease and underscoring the necessity of reconsidering malignancy when unexplained radiological abnormalities persist despite an unrevealing initial diagnostic work-up.

## Case presentation

Initial presentation and assessment

A woman in her late 60s presented to the Accident and Emergency Department with a one-day history of retrosternal chest tightness. The pain was dull, non-radiating, and unrelated to breathing, movement, or exertion. She also reported an intermittent productive cough with white sputum for several weeks, predominantly nocturnal, and approximately 4 stones (approximately 25 kg) of unintentional weight loss over the preceding seven months. She denied fever, night sweats, haemoptysis, haematuria, epistaxis, or recent change in bowel habits. She had no recent travel history.

Her past medical history included hypothyroidism and bilateral hip replacement. She had experienced two recent episodes of chest infection, both treated in the community. Her medications were levothyroxine and a statin. She was otherwise independent and well. She had never smoked tobacco and had no passive smoke exposure. There was no history of occupational lung exposures. There was no family history of lung cancer or autoimmune disease, although her father had a history of oropharyngeal cancer.

On examination, she was haemodynamically stable, afebrile, and maintaining normal oxygen saturation on room air. Her initial oxygen saturation was 96%, blood pressure 130/86, heart rate 82, and temperature 37.2°C. Chest auscultation was clear. There were no clinical features of connective tissue disease or deep vein thrombosis.

Initial investigations and differential diagnosis

Initial blood tests demonstrated a markedly elevated CRP of 155 mg/L, with normal white cell count, neutrophil count, and procalcitonin. Renal and liver function were otherwise unremarkable. The electrocardiogram showed sinus rhythm, and troponin was within the normal range. Chest X-ray on admission demonstrated diffuse parenchymal nodular changes within both lungs (Figure 1).

**Figure 1:**
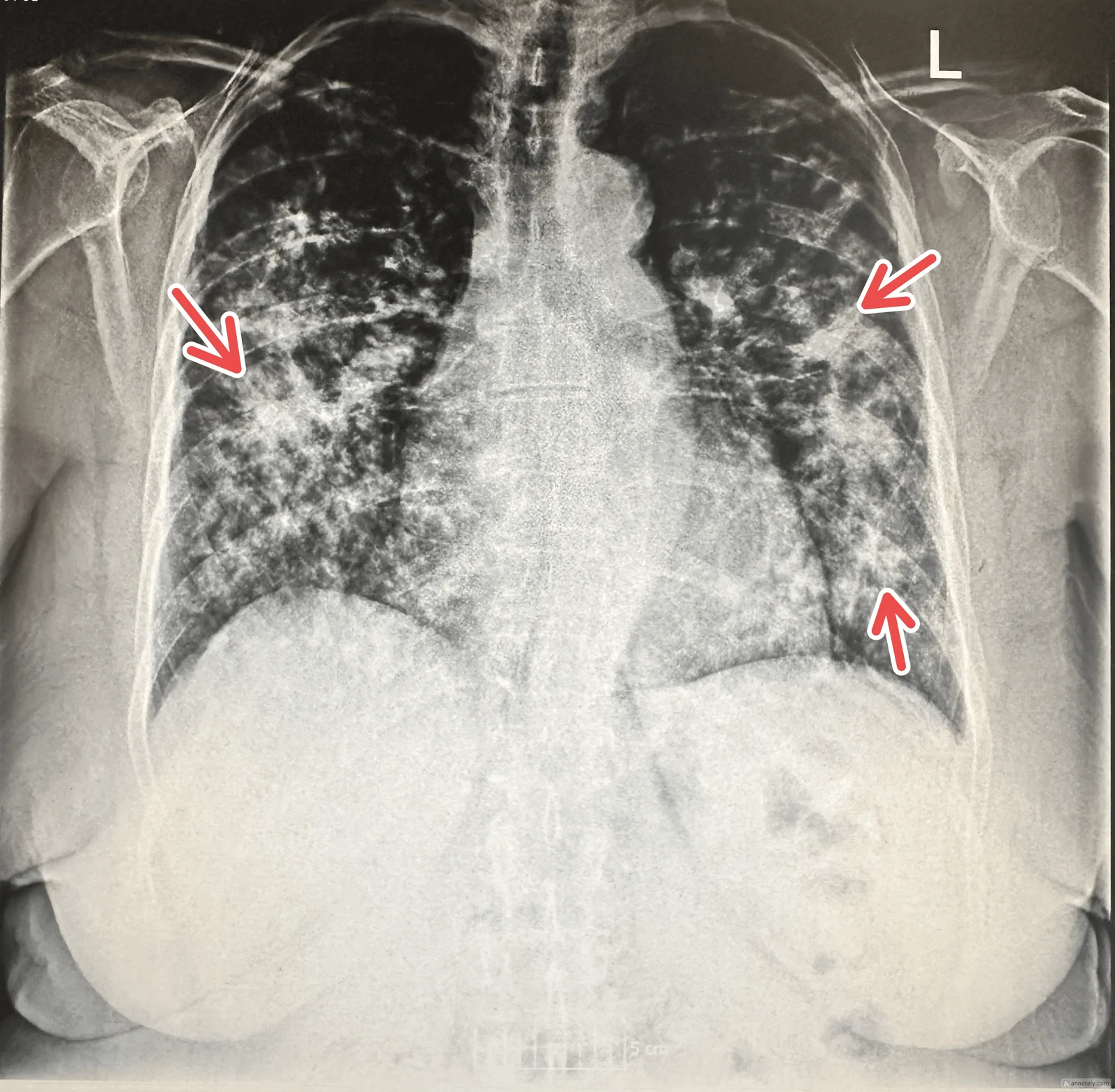
Initial chest radiograph on admission. Initial chest radiograph on admission showing diffuse bilateral parenchymal nodular opacities (red arrows), predominantly in a perihilar distribution, with associated local architectural distortion. Cardiomediastinal contours are unremarkable.

Initial blood test results are summarised in Table 1.

**Table 1:** Initial laboratory investigations on admission CRP: C-reactive protein; eGFR: estimated glomerular filtration rate; ALT: alanine aminotransferase; ALP: alkaline phosphatase

Parameters	Initial values	Normal values
Haemoglobin	136 g/L	130-170 g/L
White cell count	5.49 x 10^9^/L	4.00-11.0 x 10^9^/L
Platelet	218 x 10^9^/L	150-400 x 10^9^/L
Lymphocyte	1.62 x 10^9^/L	0.9-4.0 x 10^9^/L
Neutrophils	3.22 x 10^9^/L	1.70-7.50 × 10^9^/L
Eosinophils	0.08 × 10^9^/L	0.04 to 0.4 x 10^9^/L
CRP	155 mg/L	0-5 mg/L
Procalcitonin	0.04	<0.1 ng/mL
Sodium	134	133-146 mmol/L
Potassium	3.9	3.5-5.3 mmol/L
Urea	3.9	2.5-7.8 mmol/L
Creatinine	60	59-104 µmol/L
eGFR	90	>90 mL/min/1.73 m^2^
Albumin	45	35-50 g/L
Calcium	2.49	2.2-2.6 mmol/L
ALT	18	<50 IU/L
ALP	61	30-130 IU/L
Bilirubin	21	0-21 µmol/L
Troponin	4.7 ng/L	<11.6 ng/L

Subsequent contrast-enhanced CT of the thorax, abdomen, and pelvis demonstrated widespread bilateral, multilobar patchy and irregular areas of pulmonary consolidation, with internal cavitary change and associated tractional distortion of the fissures and airways. Areas of coarse reticular fibrotic change and architectural distortion were present within the affected lung parenchyma, with multiple small pulmonary nodules scattered throughout the surrounding lung fields. A single enlarged left mediastinal lymph node was also identified (Figure 2). The initial radiological report considered the appearances atypical for malignancy or metastatic disease and suggested granulomatous disease, including tuberculosis or sarcoidosis, and atypical infection.

**Figure 2:**
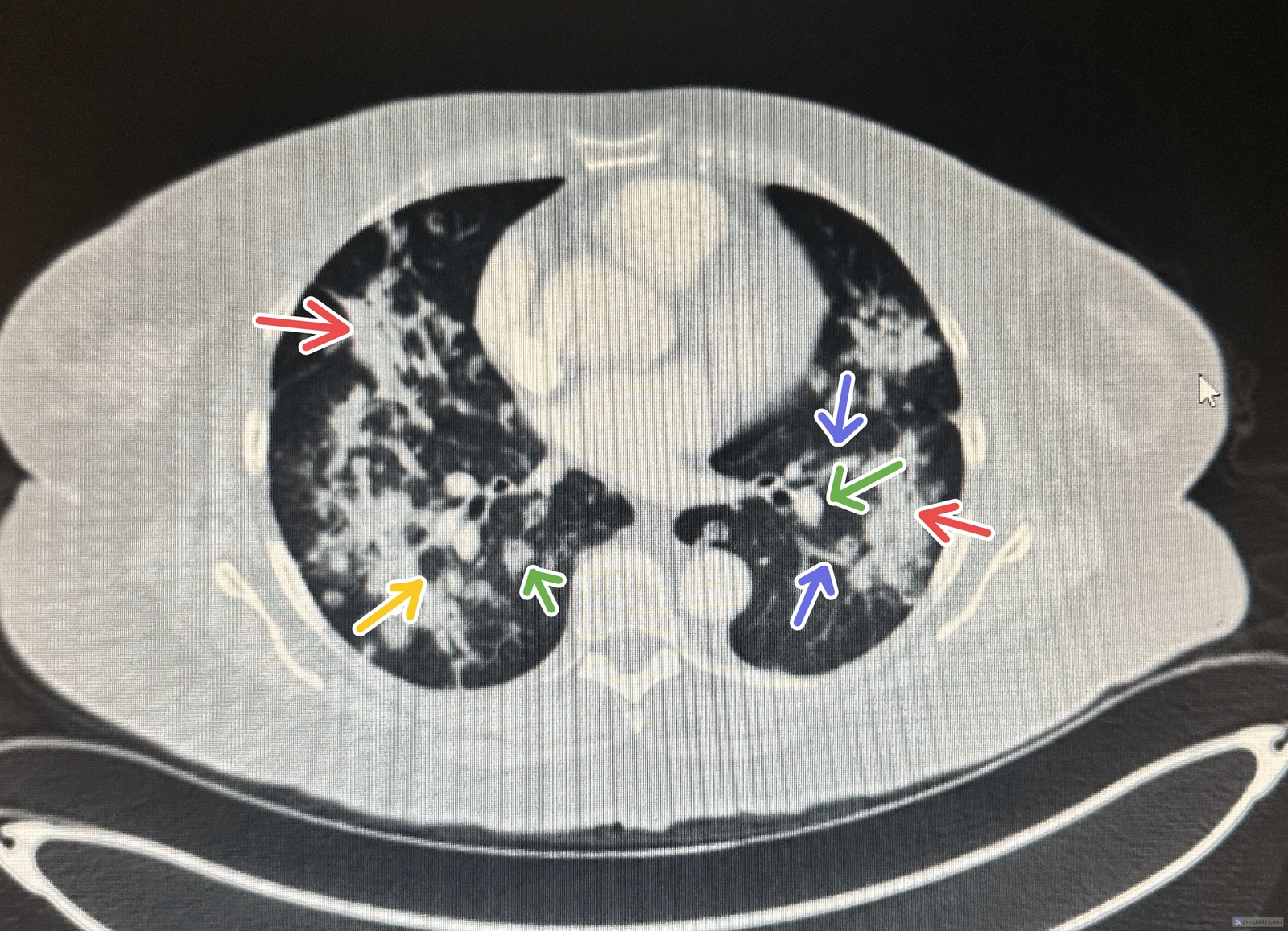
Contrast CT scan of the thorax, abdomen, and pelvis. Contrast-enhanced thoracic CT demonstrating widespread bilateral pneumonic-type involvement by invasive mucinous adenocarcinoma. Large, patchy areas of consolidation involve multiple lobes bilaterally (red arrows), with small internal lucencies consistent with cavitary change and associated tractional airway distortion (yellow arrow). Blue arrows highlight areas of marked parenchymal architectural distortion, coarse reticular fibrotic change, and traction bronchiolectasis. Multiple small, discrete satellite pulmonary nodules are scattered throughout the surrounding lung parenchyma (green arrows). Multilobar involvement and left mediastinal lymphadenopathy were demonstrated on the complete thoracic acquisition.

The respiratory team initially considered sarcoidosis the leading differential diagnosis and arranged further investigations, including serum angiotensin-converting enzyme level, vasculitis and autoimmune screens, T-SPOT.TB, and beta-D-glucan, with plans for outpatient follow-up. She was discharged with a course of oral co-amoxiclav while awaiting lung multidisciplinary team (MDT) review. Following the lung MDT review, fungal infection was considered the leading differential diagnosis at that stage, and no definite radiological features were considered diagnostic of malignancy. MDT recommended HIV, *Aspergillus* antigen (galactomannan), and additional fungal markers, along with a follow-up chest X-ray in one month.

Autoimmune workup, sarcoidosis evaluation (serum angiotensin-converting enzyme), and infectious screening (including T-SPOT.TB and HIV) were otherwise unremarkable. Subsequent bronchial wash and bronchoalveolar lavage (BAL) yielded no microbiological evidence of bacterial, mycobacterial, or fungal infection on the reported microscopy and cultures. However, Aspergillus galactomannan testing could not be performed due to insufficient sample volume. P-ANCA was positive; however, MPO and PR3 antibodies were negative, and the overall clinical and serological findings did not support ANCA-associated vasculitis. 

The autoimmune screen and serum ACE level are summarised in Table 2, while the microbiological investigations are summarised in Table 3.

**Table 2:** Autoimmune and granulomatous investigations. ACE: angiotensin-converting enzyme; ANA: antinuclear antibody; ANCA: antineutrophil cytoplasmic antibody; ESR: erythrocyte sedimentation rate; MPO: myeloperoxidase; PR3: proteinase 3; C3: complement component 3; C4: complement component 4

Investigations	Results	Interpretation
ESR	48 (1-35 mm/hr)	Mildly elevated; non-specific marker of inflammation
ANA	Negative	-
P-ANCA	Positive	Positive ANCA, negative MPO and PR3
MPO	<0.2 (0–3.4)	Negative
PR3	<0.2 (0–1.9)	Negative
Rheumatoid factor	<10.0 (LT)	Negative
Complements	C3- 1.51, C4-0.27	C3: 0.90 - 1.80 g/L C4 : 0.10 - 0.40 g/L
Immunoglobulins subclasses	Normal	-
Serum ACE	30	16-85 IU/L

**Table 3:** Microbiological and bronchoscopic investigations AFB: acid-fast bacilli; BAL: bronchoalveolar lavage; HIV: human immunodeficiency virus; TB: tuberculosis; Ag: antigen; T-SPOT.TB: tuberculosis-specific interferon-gamma release assay

Investigations	Results
Blood culture	No growth
Sputum microscopy and culture	No growth
Three sputum samples for AFB microscopy and mycobacterial culture	*Mycobacterium* not isolated in any of the three samples
T-spot TB	Negative
Beta-D-glucan	<7.0 pg/ml (Cut-off <7.0 pg/ml)
Beta-D-glucan Ag	Not detected
HIV 1 and 2 antigen/antibody	Not detected
Galactomannan	Not tested due to insufficient sample
Bronchial wash bacterial culture	No growth
Bronchial wash cytology	Bronchial cells, alveolar macrophages, mature squamous cells, and scattered lymphocytes and neutrophils. No evidence of malignancy is seen, and no granulomatous inflammation is noted.
Bronchoalveolar lavage (BAL)	Microscopy, culture, and extended fungal cultures negative

Persistent radiological abnormalities and diagnostic evaluation:

Bronchoscopy was subsequently performed because of the persistent radiological abnormalities. Bronchial wash cytology showed no malignant cells or granulomatous inflammation. BAL microscopy and bacterial, mycobacterial, and fungal cultures were negative (Table 3).

Repeat chest radiography one month later showed persistent multifocal bilateral pulmonary opacities without significant interval improvement, with possible mild progression of right-sided nodularity and right lower paratracheal lymphadenopathy (Figure 3).

**Figure 3:**
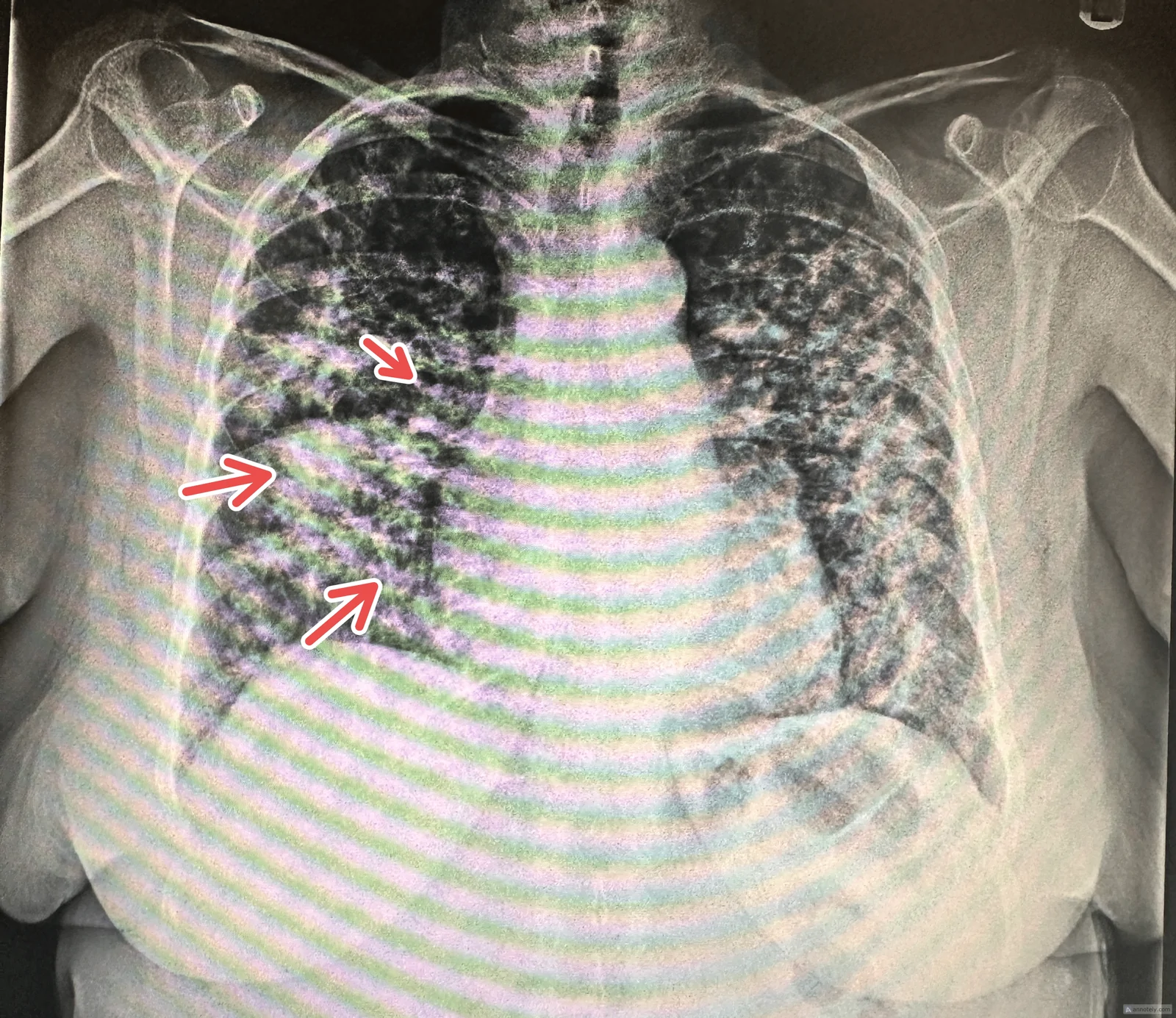
Repeat chest radiograph one month after initial presentation. Repeat chest radiograph demonstrating persistent multifocal areas of bilateral pulmonary consolidation, with no significant interval change compared with the initial radiograph. Red arrows indicate areas of increased right-sided nodularity and the right lower paratracheal lymph node.

Definitive diagnosis

Given the persistent radiological abnormalities and non-diagnostic infectious and bronchoscopic investigations, the lung MDT recommended CT-guided biopsy. Two months after her initial presentation, CT-guided biopsy of the lung lesion demonstrated a focus of moderately differentiated invasive adenocarcinoma with mucinous differentiation. The biopsy background showed patchy chronic inflammatory infiltrates, pigment-containing intra-alveolar macrophages, and mild respiratory epithelial hyperplasia. Tumour cellularity in the sampled area was approximately 60%. Immunohistochemistry demonstrated diffuse CK7 positivity, negative CK20 expression, and focal nuclear TTF-1 positivity, with the overall findings supporting a primary pulmonary origin (Table 4).

**Table 4:** Histopathological and immunohistochemical gindings CK7: cytokeratin 7; CK20: cytokeratin 20; TTF-1: thyroid transcription factor-1

Investigations	Findings
Histopathology	Moderately differentiated invasive adenocarcinoma with mucinous differentiation. The biopsy showed patchy chronic inflammatory infiltrates, pigment-containing intra-alveolar macrophages, and mild respiratory epithelial hyperplasia. Tumour cellularity in the sampled area was approximately 60%.
Immunohistochemistry	CK7: diffuse positive; CK20: negative; TTF-1: focal nuclear positive. Overall findings support a primary pulmonary origin.

Staging and treatment

Following the diagnosis of moderately differentiated IMA of the lung, she was referred to oncology. The lung MDT staged the disease as cT4N3M1a (stage IVA) based on the overall radiological distribution and pneumonic-type presentation of the IMA. Initial thoracic CT demonstrated widespread patchy consolidations with cavitation, traction on the fissures, and multiple pulmonary nodules involving multiple lobes of both lungs, without a single dominant primary lesion. The extensive multilobar pulmonary involvement was considered by the MDT in assigning the T4 descriptor, while tumour involvement of both lungs was interpreted as M1a intrathoracic metastatic disease. Regarding nodal staging, initial imaging demonstrated left mediastinal lymphadenopathy, and follow-up CT identified a right lower paratracheal lymph node (station 4R). In the context of the diffuse, non-lateralised pulmonary disease, the distribution of mediastinal nodal involvement on both sides was interpreted by the MDT as N3 disease. Accordingly, the final MDT stage was cT4N3M1a (stage IVA).

Molecular testing was requested to guide systemic therapy, particularly given the patient’s lifelong non-smoking status and the possibility of an actionable driver alteration. Molecular evaluation included plasma circulating tumour DNA (ctDNA) next-generation sequencing using FoundationOne Liquid CDx and tissue-based PD-L1 immunohistochemistry. No clinically actionable driver alteration was identified on the reported plasma circulating tumour DNA (ctDNA) assay. However, the ctDNA tumour fraction was <1%, which may have reduced the sensitivity of plasma-based genomic testing; therefore, an alteration below the assay's detection threshold cannot be excluded. PD-L1 expression was negative (tumour proportion score [TPS] 0%). A TP53 variant was detected at a very low variant allele frequency (0.2%) but was not considered actionable for targeted therapy.

Table 5 summarises the detailed molecular testing results.

**Table 5:** Molecular testing and tumour biomarker profile ctDNA: circulating tumour DNA; NGS: next-generation sequencing; TP53: tumour protein p53; bTMB: blood tumour mutational burden; Mb: megabase; MSS: microsatellite stable; PD-L1: programmed death-ligand 1; TPS: tumour proportion score; TC: tumour cells; IC: immune cells

Test	Method/assay	Key findings	Interpretation
ctDNA NGS	FoundationOne Liquid CDx (324 genes; including EGFR, ALK, BRAF, KRAS, MET, ROS1, RET, and NTRK1–3)	No actionable alterations were identified in the tested driver genes (EGFR, ALK, BRAF, KRAS, MET, ROS1, RET, NTRK1–3). TP53 variant detected.	No clinically actionable driver alteration identified
TP53 variant	ctDNA NGS	TP53 c.710T>A p.(Met237Lys), variant allele frequency 0.2%	TP53 alteration identified; not considered actionable for targeted therapy in this setting
Tumour fraction (ctDNA)	FoundationOne Liquid CDx	<1%	Low tumour fraction; limits sensitivity of plasma-based testing
Blood tumour mutational burden (bTMB)	FoundationOne Liquid CDx	4 mutations/Mb	Low/intermediate; not independently treatment-directing
Microsatellite status	FoundationOne Liquid CDx	Microsatellite stable (MSS)	No evidence of microsatellite instability
PD‑L1 (SP263)	Immunohistochemistry	TPS 0%	PD‑L1 negative.
PD‑L1 (SP142)	Immunohistochemistry	TC 0%, IC 0%	PD‑L1 negative

Five months after her initial presentation, following oncology MDT review and completion of molecular testing, she commenced first-line carboplatin, pemetrexed, and pembrolizumab. Four cycles were planned. This regimen was selected in the absence of an identified actionable driver alteration on the reported molecular testing and in the setting of PD-L1-negative disease, with treatment guided by established approaches for advanced non-squamous NSCLC. However, the low ctDNA tumour fraction (<1%) may have reduced the sensitivity of plasma-based testing, and an alteration below the assay’s detection threshold cannot be excluded.

Follow-up and outcome

She remains under oncology follow-up and is continuing her planned chemo-immunotherapy. At the most recent oncology review, she remained clinically stable and was tolerating treatment without dose-limiting toxicity. She is awaiting CT imaging after completion of her treatment to assess response.

## Discussion

IMA is an uncommon histological subtype of lung adenocarcinoma with recognised clinical, radiological, pathological, and molecular characteristics that may differ from those of non-mucinous adenocarcinoma. Radiologically, IMA may present as multifocal pneumonia-like consolidation, air-space opacities, nodules, or ground-glass changes [1,7]; cavitation is less typical but has been reported [2,8]. These features can complicate diagnosis because they may resemble infectious or inflammatory lung disease. Diagnosis was more challenging in our patient because the cavitation further broadened the radiological differential. Extensive infectious, autoimmune and granulomatous screening tests could not identify any alternative aetiology, and the pulmonary abnormalities persisted despite initial treatment. This case highlights how an atypical, persistent radiological presentation can delay recognition of IMA and emphasises the importance of reconsidering the differential diagnosis and obtaining a tissue diagnosis when abnormalities persist despite initial diagnostic work-up.

IMA has been described radiologically as having solitary and pneumonic-type phenotypes [7]. In addition, IMA can present as air-space opacities, including ground-glass and consolidation opacities, and solid nodules, with some reports describing a crazy-paving appearance, although this pattern is not specific to IMA [7]. Cavitation may be an uncommon finding in IMA [2,8]. Our patient presented with widespread consolidations, cavitation, and fibrotic and tractional changes. These features initially raised the possibility of sarcoidosis and atypical infection. A normal serum angiotensin-converting enzyme level and the absence of a typical pattern of thoracic lymphadenopathy did not provide supportive evidence for sarcoidosis; however, these findings alone could not exclude the diagnosis. Sarcoidosis was subsequently considered less likely based on the overall clinical, radiological, microbiological, and histopathological assessment. The presence of productive cough, elevated C-reactive protein, and extensive bilateral radiological abnormalities initially supported an infectious or inflammatory process. In contrast, no infectious cause was identified on extensive microbiological investigations, and persistent clinical symptoms and radiological abnormalities despite antibiotic treatment reduced the likelihood of an untreated infectious process. Furthermore, vasculitis screening tests were also performed because of the presence of atypical multifocal consolidation and a history of weight loss with non-specific symptoms. Although P-ANCA was positive, MPO and PR3 antibodies were negative, with preserved renal function and no other accompanying clinical features of systemic vasculitis. Therefore, the isolated P-ANCA positivity, in the absence of MPO or PR3 antibodies, compatible clinical manifestations, or other supportive organ involvement, was considered insufficient to support a diagnosis of ANCA-associated vasculitis.

The patient's lifelong non-smoking status, together with widespread bilateral multifocal consolidation, nodules, cavitation, and fibrotic or tractional changes rather than a dominant focal mass, may have contributed to the initial consideration of non-malignant differentials. This is notable because a substantial proportion of patients in some IMA cohorts are never-smokers; in one retrospective study of 91 screening-detected IMAs, 73.6% of patients had never smoked [9]. Conversely, the marked weight loss, recurrent chest infections, persistent symptoms and radiological abnormalities despite antibiotics represented red flags for an underlying malignant process and supported the need for diagnostic reassessment.

Both bronchial washing and BAL were non-diagnostic, revealing bronchial epithelial cells, alveolar macrophages, and scattered lymphocytes and neutrophils, but no malignant cells or granulomatous inflammation. Given the predominantly parenchymal and multifocal nature of the abnormalities, the non-diagnostic bronchoscopic findings did not establish an alternative diagnosis or exclude an underlying malignancy. Furthermore, IMA may present diagnostic challenges in cytological samples because mucin-containing tumour cells can show relatively inconspicuous cytological atypia [10]. Given the persistent radiological abnormalities and unresolved diagnostic uncertainty, a CT-guided biopsy of the lung was subsequently performed, which ultimately confirmed the diagnosis of IMA. The atypical radiological presentation and initial diagnostic uncertainty therefore contributed to the delay in establishing the diagnosis.

Importantly, the CT-guided biopsy established the histological diagnosis and enabled definitive diagnostic classification. Given the patient's lifelong non-smoking status and the recognised molecular heterogeneity of IMA, molecular testing was performed to assess for potentially actionable genomic alterations [3,11]. Plasma-based comprehensive genomic profiling using FoundationOne Liquid CDx did not identify any clinically actionable alteration among the genes assessed, and PD-L1 expression was negative. However, the ctDNA tumour fraction was <1%, which reduces confidence in a negative plasma-based result and limits its ability to exclude an underlying actionable genomic alteration [12]. As tissue-based comprehensive genomic sequencing or dedicated fusion testing was not performed, an undetected actionable alteration cannot be excluded. Therefore, the absence of an actionable alteration on plasma-based testing should be interpreted cautiously and does not definitively exclude an actionable genomic driver. In the absence of an identified actionable driver alteration, treatment was selected according to established systemic treatment approaches for advanced non-squamous non-small cell lung cancer (NSCLC).

Evidence specifically guiding systemic treatment for advanced IMA remains limited [13]. The KEYNOTE-189 trial showed significantly improved overall and progression-free survival with pembrolizumab plus pemetrexed and platinum-based chemotherapy compared with chemotherapy alone in previously untreated metastatic non-squamous NSCLC without EGFR or ALK alterations [14]. Although IMA was not specifically studied as a separate histological subgroup, these data provide a rationale for the regimen used in this patient. However, responses to systemic therapy in IMA may differ from those observed in conventional non-mucinous adenocarcinoma, and evidence specific to advanced IMA remains limited.

A limitation of this case is the low ctDNA tumour fraction (<1%), which reduces the confidence in a negative plasma-based result and may limit the detection of genomic alterations [12]. Although plasma-based comprehensive genomic profiling did not identify an actionable driver alteration, the possibility of an alteration below the assay's detection threshold cannot be excluded with certainty [12]. Furthermore, tissue-based comprehensive genomic sequencing or dedicated fusion testing was not performed. In addition, at the time of manuscript preparation, post-treatment imaging had not yet been performed; therefore, treatment response could not be assessed. Another important limitation of this case report is that the original histopathological and immunohistochemistry images were not available to the authors for inclusion at the time of manuscript preparation, despite attempts to obtain them. The diagnosis was therefore based on the formal histopathology and immunohistochemistry reports obtained.

## Conclusions

This case demonstrates a relatively unusual radiological presentation of IMA with diffuse bilateral consolidative and nodular changes, cavitation, and fibrosis mimicking granulomatous disease or atypical infection. It highlights the importance of considering malignancy in the differential diagnosis when warning features, such as significant weight loss or recurrent episodes of clinically diagnosed chest infection, are present, even in lifelong never‑smokers, particularly when initial investigations are negative. Image-guided tissue diagnosis can play a pivotal role in establishing the definitive diagnosis when microbiological, autoimmune, and bronchoscopic investigations fail to identify the underlying aetiology. This case also highlights the role of molecular testing in evaluating for potentially actionable alterations in IMA. However, a negative plasma-based molecular profile, particularly with a low ctDNA tumour fraction, does not definitively exclude an underlying actionable genomic alteration. Treatment should therefore be guided by multidisciplinary assessment, stage, histology, and the available molecular results, recognising that IMA-specific evidence remains limited. 
